# Hodgkin's disease and birth outcome: a Danish nationwide cohort study

**DOI:** 10.1038/sj.bjc.6604126

**Published:** 2007-12-04

**Authors:** V Langagergaard, E Horvath-Puho, M Nørgaard, B Nørgård, H T Sørensen

**Affiliations:** 1 Department of Clinical Epidemiology, Aarhus University Hospital, Ole Worms Allé 150, Aarhus C DK-8000, Denmark; 2 Department of Epidemiology, Institute of Public Health, Aarhus University, Vennelyst Boulevard 6, Aarhus C DK-8000, Denmark

**Keywords:** Hodgkin's disease, epidemiology, pregnancy, birth outcome, cohort study

## Abstract

In a Danish nationwide cohort study of 292 births from 1973 to 2002 in women with Hodgkin's disease (HD), we compared birth outcome with 14 042 births from a cohort of mothers without cancer. We found no substantially increased risk of preterm birth, low birth weight at term, or stillbirth and no difference in proportion of male newborns for 192 children of women with HD before pregnancy. The prevalence odds ratio (POR) for congenital abnormalities was 1.7 (95% confidence interval (CI): 0.9–3.1). Among 15 newborns of mothers diagnosed during pregnancy, the POR of preterm birth was 26.6 (95% CI: 8.5–83.0), but five out of the eight preterm deliveries among these women were elective. We found no substantially increased risk of adverse birth outcome among 85 newborns of women diagnosed within 2 years postpartum, though effect estimates were imprecise. The overall findings are reassuring, they cannot exclude the possibility of an increased risk of congenital abnormalities for newborns of women diagnosed with HD before pregnancy.

Hodgkin's disease (HD) can affect women of childbearing age (Fisher and Hancock, 1996). Advances in its treatment have led to an overall 5-year relative survival of more than 80% (Melbye and Adami, 2002). However, there is concern (Swerdlow *et al*, 1996) that treatment may affect future pregnancies either by direct effects on the reproductive tract or by causing mutations in germ cells (Nagarajan and Robison, 2005). Furthermore, cancer treatment administered in the first trimester may be teratogenic (Fisher and Hancock, 1996), while detriments in maternal well-being may influence pregnancies in women with preclinical HD (Koren *et al*, 1996).

Nevertheless, data concerning birth outcome in women with previous HD are sparse and consist mainly of case series (McKeen *et al*, 1979; Andrieu and Ochoa-Molina, 1983; Green and Hall, 1988; Aisner *et al*, 1993; Brierley *et al*, 1998). A few of these case series, which included birth outcome in 15–54 women found a high prevalence of adverse outcome. Green and Hall (1988) reported 4 stillbirths among 28 pregnancies (14.3%) in women with previous HD, while McKeen *et al* (1979) reported 6 premature/low birth weight children (15.0%) and 3 with major congenital abnormalities (7.5%) among 40 pregnancies. The remaining studies found little, if any, detrimental effect on birth outcome among women capable of becoming pregnant (Andrieu and Ochoa-Molina, 1983; Aisner *et al*, 1993; Brierley *et al*, 1998). Likewise, a few case series found normal birth outcome in women diagnosed with HD during or shortly after pregnancy (Woo *et al*, 1992; Anselmo *et al*, 1999; Aviles and Neri, 2001).

We examined the risk of adverse birth outcomes in a Danish nationwide cohort of women with HD before or during pregnancy, or within 2 years after delivery and compared them with those in a cohort of pregnant women without cancer.

## MATERIALS AND METHODS

We used the Danish Cancer Registry, which has covered all incident cancers in Denmark since 1943, classified according to the International Classification of Diseases (ICD-7) (Storm *et al*, 1997), to trace all women with a diagnosis of HD (ICD-7 code 201). Information included the civil registration number of the woman, date of diagnosis, and radiation treatment administered within 4 months of diagnosis.

Since 1 January 1973, all births in Denmark have been registered in the Danish Medical Birth Registry (Knudsen and Olsen, 1998). Data are obtained from birth notifications, which are completed by midwives (who attend all births, including home births, in Denmark). The main variables in the Birth Registry are gestational age, birth weight, parity, stillbirth, place of birth, and the civil registration number of the mother and child (which encodes sex and date of birth and is assigned to all live-born children and new residents; Frank, 2000).

Using the civil registration number, we linked the Cancer Registry data with the Birth Registry to establish a cohort of all Danish women with a diagnosis of HD in, 1970–2002, and who gave birth in 1973–2002. Women were included if they were diagnosed with HD before pregnancy, during the pregnancy, or until 2 years postpartum. We restricted all analyses to singleton births, since multiple births have been associated with an adverse birth outcome (Pinborg *et al*, 2004).

For each birth by a woman with HD, 50 comparison births matched by month and year of the birth, by county of mother's residence, and born to 50 different women who were not diagnosed with any cancer before, during, or within 2 years after the pregnancy were selected from the Birth Registry. If fewer than 50 births fulfilled the matching criteria, we used all the available births. If more than 50 comparison births were eligible after matching, we selected a random subset of 50 births. On average, 48 comparison births were selected for each exposed birth.

The outcome data collected from the Birth Registry included preterm birth (birth before 37 completed weeks of pregnancy), low birth weight at term <2500 g with ⩾37 completed weeks), stillbirth (delivery of a dead foetus at ⩾28 completed weeks of pregnancy), male proportion of newborns, and birth weight. The potential confounders included maternal age, parity, gestational age, and calendar period of the birth. For live-born children, data on congenital (including chromosomal) abnormalities, diagnosed during the first year of life were collected from the National Hospital Discharge Registry, covering all hospital discharge diagnoses since 1977 and outpatient visits since 1995 (Andersen *et al*, 1999). Thus, data on congenital abnormalities applied to births from 1977 to 2002. The data include the civil registration number, dates of admission and discharge, and up to 20 discharge diagnoses, (ICD-8 before 1994 and ICD-10 from 1994 onwards; Andersen *et al*, 1999). The codes for congenital (including chromosomal) abnormalities were 740.00–759.99 in ICD-8 and Q0.00 to Q99.9 in ICD-10. Diagnoses of congenital dislocation of the hip and undescended testis were excluded because of their poor validity (Larsen *et al*, 2003).

Birth weights ⩾7000 g probably reflected coding errors and were excluded, as were births with a gestational age below 20 or over 44 weeks. Owing to a coding change in the Birth Registry in 1978, there were more missing data on gestational age for the years 1978–1981 than for other years (mean 22.6% missing for 1978–1981, compared with 0.8% in 1973–1977 and 1.2% in 1982–2002). Births without data on gestational age were excluded from the study (*N*=20 in the exposed and 698 in the comparison cohort).

We classified the births of women with HD into three groups: group 1 included the first birth after an HD diagnosis (that is, women who were diagnosed before pregnancy). Group 2 included the births by women diagnosed with HD during pregnancy (that is, diagnosed between the first day in the last menstruation until the date of birth). Group 3 included births by women who were diagnosed with HD after delivery (that is, diagnosed between the day after the delivery until 2 years later). If a woman gave birth more than once in this 2-year period, only the last birth before the HD diagnosis was included based on the assumption that the preclinical cancer would be more likely to affect the birth closest to the time of diagnosis.

For all three groups, we computed the difference between proportions of male newborns of mothers with HD and comparison mothers.

We computed prevalence odds ratios (PORs) as estimates of the relative risks with associated 95% confidence intervals (95% CIs) for preterm birth, low birth weight at term, stillbirth, and congenital abnormalities. The PORs were controlled for month and year of birth and county of mother's residence by matching. We used unconditional logistic regression analysis to further adjust for maternal age and parity. We also included the calendar period of the birth (1973–1986, 1987–1994, and 1995–2002), as an independent variable in the model. Although there was no change in the risk estimates when calendar period of birth was included in the model, we kept the variable in the model. Stillborn children were excluded from the analyses of preterm birth, low birth weight at term, and congenital abnormalities.

To examine whether sex of the child or maternal radiotherapy modified the POR estimates for births in group 1, we repeated the analyses in strata of boys and girls and strata of births of women who were treated with radiotherapy and women who were not. Furthermore, to examine whether calendar period of HD diagnosis modified the POR estimates for births in group 1, we repeated the analyses in different calendar periods of HD diagnosis (1981–1990 and 1991–2000), using 1970–1980 as reference. We used the Wald test to evaluate the homogeneity of the POR estimates for congenital abnormalities in 1981–1990 and 1991–2000. The low count of outcome events in groups 2 and 3 precluded stratified analyses.

We used linear regression to estimate differences in mean birth weight, while controlling for maternal age, parity, gestational age, and calendar period of birth. Stillborn children were excluded from these analyses.

The study was approved by the Danish Data Protection Agency (record no. 2003-41-2833). All analyses used SAS software, version 8.2. The SAS procedures used were PROC FREQ, PROC MEANS, PROC LOGISTIC, and PROC GLM.

## RESULTS

In total, we identified 292 singleton births delivered by women with HD and selected 14 042 singleton births for the comparison cohort. The characteristics of births in the three groups and their comparison births are shown in Table 1. Of the 292 births by women with HD, 192 occurred in group 1. The median number of days from the time of diagnosis until pregnancy (that is, the first day in the last menstruation) was 1824 days (range: 279–7877 days). The majority of women (76%) in group 1 were ⩾20 years of age at time of HD diagnosis (data not shown) and the mean age at delivery was 29.0 years. Seventy percent of the women gave birth for the first time. Group 2 included 15 births (eight women were diagnosed in the second trimester and seven in the third). In this group, the mean age at delivery was 26.5 years and 66.7% gave birth for the first time. Group 3 included 85 births. The median number of days from date of giving birth until date of cancer diagnosis was 321 days (range: 6–709 days). The mean age at delivery was 28.0 years, and 49.4% of the women gave birth for the first time.

The prevalence of male newborns of women with HD in group 1 was 50.0%, compared with 51.3% among the matched comparison mothers (difference=−1.3%, 95% CI: −8.4 to 5.8). The corresponding findings were 73.3 *vs* 50.1% (difference=23.2%, 95% CI: 5.1–45.6) for group 2, and 61.2 *vs* 51.4% (difference=9.8%, 95% CI: −0.7 to 20.3) for group 3.

Table 2 shows PORs for preterm birth, low birth weight at term, stillbirth, and congenital abnormalities for newborns in all three groups. For group 1, there was no increased risk of preterm birth or low birth weight at term. We found only 1 stillbirth among 192 births, corresponding to a POR of 2.0 (95% CI: 0.3–15.4). The POR for congenital abnormalities was 1.7 (95% CI: 0.9–3.1). In groups 2 and 3, there were no children with low birth weight at term and no stillbirths. The POR of preterm birth in group 2 was 26.6 (95% CI: 8.5–83.0). However, five of the eight preterm deliveries among women with HD were elective preterm deliveries. There was 1 child with a congenital abnormality among 13 births in group 2 (POR=2.7, 95% CI: 0.3–22.8) and 4 children with congenital abnormalities among 78 births in group 3 (POR=1.6, 95% CI: 0.6–4.5). The specific types of congenital abnormalities identified in children of women with HD in groups 1, 2, and 3 are listed according to affected organ system in Table 3.

Table 4 shows the birth outcomes in group 1, stratified according to maternal radiotherapy (yes/no) and three calendar periods of HD diagnosis. Stratification suggested a slightly lower risk (except for stillbirths) of adverse birth outcomes in women who had received radiotherapy. Furthermore, we found that the POR for congenital abnormalities increased with calendar time of HD diagnosis (Wald test of the homogeneity of the POR estimates for 1981–1990 and 1991–2000, with 1970–1980 as reference; *P*=0.25). Stratification according to sex of newborns did not substantially change the estimates (data not shown).

The multiple linear regression analyses showed that newborns in all three groups had nearly the same mean birth weight as newborns in the comparison cohort (data not shown).

## DISCUSSION

This nationwide cohort study on the relation between maternal HD and adverse birth outcome did not show any increased risk of preterm birth or low birth weight at term, and no substantial increased risk of stillbirth in women with previous HD. However, we cannot rule out the possibility of a higher risk of congenital abnormalities for newborns of these women.

The accuracy of our risk estimates depends on several factors. The main strength of the study is the underlying uniform health-care system, with complete registration of cancers and births and complete follow-up on congenital abnormalities diagnosed during the first year of life, allowing for a population-based design. Information on congenital abnormalities in the Hospital Discharge Registry is generally of high quality, with an 85% correct coding rate (Larsen *et al*, 2003). The quality of most outcome variables in the Birth Registry is high, but gestational age is subject to some misclassification (Kristensen *et al*, 1996) but is probably non-differential between HD and cancer-free mothers.

Although our study population was large compared with other studies, a limitation is the small number of outcomes. Furthermore, the data lacked clinical detail on radiation fields, doses, and duration of treatment, and we had no information on chemotherapy or disease stage; radiotherapy details (yes/no) from the Cancer Registry may be inaccurate, because they are not routinely validated. However, a study of childhood cancer survivors reported that 97 out of 110 patients treated with radiotherapy (88%), and 78 out of 79 patients not treated with radiotherapy (99%) were correctly coded in the Registry (Ross *et al*, 2003).

Women with early-stage HD, which is often located above the diaphragm, were probably more likely than women with more advanced stages, to receive radiotherapy, since the typical treatment of early-stage disease in our study period has been either radiation alone (with minimal effect on the gonads in case of supradiaphragmatic location), or a few series of combination chemotherapy followed by radiation. In contrast, later stages of HD have typically been treated with six series of combination chemotherapy and only rarely radiotherapy. Thus, an uneven distribution of stage could have biased our results and may explain our finding of a lower risk of adverse birth outcomes for women treated with radiotherapy, compared with those who were not.

Fetal abnormalities may lead both to miscarriage (Yusuf and Naeem, 2004) and to induced abortion, but we had no data on these outcomes. Thus, selection bias could have occurred if women with HD had more miscarriages and induced abortions related to fetal abnormalities than did comparison mothers. Such bias would lead us to underestimate the risk of congenital abnormalities in newborns of women with HD.

It has been suggested that mutagenic exposure of germ cells (that is, chemotherapy or radiation) may decrease the proportion of male newborns in female survivors of cancer due to sex-linked lethal mutations (Nagarajan and Robison, 2005). Our data, however, showed no substantial decrease in the male proportion of newborns in group 1, indicating that earlier treatment for HD is not a risk factor for early male abortion. For newborns in group 2, there was an increase in the male proportion compared with newborns of comparison mothers. This finding is surprising and may be due to chance. The male proportion of newborns in Denmark is approximately 51.2% (Hansen *et al*, 1999).

We believe that our study is the first to estimate relative risks for congenital abnormalities among newborns of women with HD. The increased risk estimates found after diagnoses during or shortly after pregnancy were imprecise. However, it is relevant that teratogens increase the rate of specific abnormalities but not all abnormalities (Mitchell, 2000), and we were unable to evaluate the risk of specific abnormalities. Small cohort studies can detect only large increases in the risk of specific congenital abnormalities and are limited in their ability to provide an assurance of safety. Our finding of a higher risk of abnormalities for newborns of women with HD from 1991 to 2000 (before their pregnancy) may be a diagnostic bias caused by a recently increased interest in such risk after maternal cancer treatment.

Overall, our findings are in line with the existing studies. Two cohort studies found no substantial increased risk of low birth weight and no congenital abnormalities among newborns of 15 women with previous HD (Janov *et al*, 1992) and no increased risk of preterm birth, low birth weight, stillbirth, congenital abnormalities, or chromosomal abnormalities among 49 children of 16 women and 11 men who had previously been treated for HD (Swerdlow *et al*, 1996). Both studies were compared with birth outcomes in the general population. Another cohort study compared 52 births of 29 women previously treated for HD with births of siblings of the women (Holmes and Holmes, 1978). There was no overall increased risk of adverse birth outcome (that is, congenital abnormalities and stillbirths combined) for HD patients and no increased risk associated with radiation treatment alone (supra- or infradiaphragmatic), whereas women treated with both chemotherapy and radiation were more likely to have an adverse birth outcome (*P*=0.047). These three studies, however, were all based on small study population and did not control for potential confounders.

Recently, a large cohort study of female survivors of childhood cancer found that 19.2% of 337 women with childhood HD had a preterm birth compared with 12.6% among sibling controls (Signorello *et al*, 2006). Another study reported 11 stillbirths among 729 births of female survivors of childhood HD, corresponding to a relative risk of 1.6 (95% CI: 0.64–4.03) (Green *et al*, 2002). We found no increased risk of preterm birth and only 1 stillbirth among 192 women, of whom more than 75% had been diagnosed with HD in adulthood (⩾20 years of age at diagnosis).

The 26-fold increased risk of a preterm delivery for women diagnosed with HD during pregnancy reflected a higher rate of elective early delivery, probably to allow an early start of cancer therapy. This finding is consistent with another study on pregnant women with HD (Smith *et al*, 2001) which identified 172 cases of HD diagnosed from 9 months preceding delivery until 12 months after delivery and found relative risks of 2.4 (95% CI: 1.6–3.5) for prematurity and 3.6 (95% CI: 1.5–8.9) for very low birth weight. The authors suggested that these findings reflected a higher rate of elective early deliveries to allow initiation of therapy. In contrast, a historical cohort study, which included 40 births of women who were pregnant between 9 months before and 3 months after their first treatment for HD, reported no increased risk of preterm birth or induced deliveries (Lishner *et al*, 1992). Furthermore, it indicated no difference in mean birth weight compared with controls and no increased risk of stillbirths, and overall, its findings corroborate our data, except for preterm births.

The overall findings of this nationwide cohort study are reassuring, but we cannot rule out the possibility of an increased risk of congenital abnormalities in offspring of women diagnosed with HD before pregnancy.

## Figures and Tables

**Table 1:** Characteristics of births by women with Hodgkin's disease and by women in the comparison cohort

	**Group 1**		**Group 2**		**Group 3**	
	**Women with Hodgkin's (*N*=192)**	**Comparison cohort (*N*=9247)**	**Women with Hodgkin's (*N*=15)**	**Comparison cohort (*N*=706)**	**Women with Hodgkin's (*N*=85)**	**Comparison cohort (*N*=4089)**
*Maternal age at delivery, number (%)*						
<25 years	27 (14.1)	1916 (20.7)	5 (33.3)	176 (24.9)	19 (22.4)	937 (22.9)
25–29 years	74 (38.5)	3528 (38.2)	7 (46.7)	284 (40.2)	36 (42.4)	1627 (39.8)
30–34 years	69 (35.9)	2628 (28.4)	2 (13.3)	175 (24.8)	22 (25.9)	1101 (26.9)
⩾35 years	22 (11.5)	1175 (12.7)	1 (6.7)	71 (10.1)	8 (9.4)	424 (10.4)
						
*Age at delivery (years)*						
Mean (±s.d.)	29.0 (±4.4)	28.6 (±4.9)	26.5 (±4.4)	28.0 (±5.0)	28.0 (±4.9)	28.2 (±4.8)
Minimum/maximum	16–38	15–47	20–36	16–45	18–41	16–46
						
*Parity, number (%)*						
1	135 (70.3)	4204 (45.5)	10 (66.7)	346 (49.1)	42 (49.4)	1848 (45.2)
⩾2	57 (29.7)	5031 (54.5)	5 (33.3)	358 (50.9)	43 (50.6)	2238 (54.8)
Data missing	0 (0.0)	12 (0.1)	0 (0.0)	2 (0.3)	0 (0.0)	3 (<0.1)
						
*Calendar period of birth, number (%)*						
1973–1986	59 (30.7)	2771 (30.0)	7 (46.7)	319 (45.2)	31 (36.5)	1472 (36.0)
1987–1994	52 (27.1)	2540 (27.4)	3 (20.0)	144 (20.4)	30 (35.3)	1458 (35.7)
1995–2002	81 (42.2)	3936 (42.6)	5 (33.3)	243 (34.4)	24 (28.2)	1159 (28.3)
						
*Offspring (sex), number (%)*						
Male	96 (50.0)	4735 (51.3)	11 (73.3)	353 (50.1)	52 (61.2)	2101 (51.4)
Female	96 (50.0)	4500 (48.7)	4 (26.7)	351 (49.9)	33 (38.8)	1985 (48.6)
Data missing^a^	0 (0.0)	12 (0.1)	0 (0.0)	2 (0.3)	0 (0.0)	3 (<0.1)
						
*Gestational age (weeks)* b						
Mean (±s.d.)	39.5 (±2.1)	39.5 (±1.9)	37.0 (±3.4)	39.6 (±1.7)	39.5 (±1.8)	39.6 (±1.8)
Minimum/maximum	26–42	23–44	33–42	23–44	31–43	25–44
						
*Birth weight (g)* b						
Mean (±s.d.)	3462 (±581)	3464 (±571)	2938 (±649)	3450 (±539)	3412 (±576)	3460 (±556)
Minimum/maximum	803–5000	655–5600	1690–4400	570–5200	1870–4720	820–5530

Abbreviation: CPR-number=civil registration number.

Group 1: births by women diagnosed with Hodgkin's disease before pregnancy.

Group 2: births by women diagnosed with Hodgkin's disease during pregnancy.

Group 3: births by women diagnosed with Hodgkin's disease within 2 years after giving birth.

a Births with missing data on sex were all stillbirths who had no CPR-number.

b Stillborn babies were excluded from the analyses of mean gestational age and mean birth weight.

**Table 2:** Prevalence odds ratios of birth outcome in women with Hodgkin's disease

	**Hodgkin's disease cohort**,	**Comparison cohort**,	**Prevalence odds ratio^a^**	**Prevalence odds ratio^b^**
	**outcome/total (%)**	**outcome/total (%)**	**(95% CI)**	**(95% CI)**
*Births in group 1*	(*N*=192)	(*N*=9247)		
Preterm birth	12/191 (6.3)	479/9162 (5.2)	1.2 (0.7–2.2)	1.1 (0.6–2.0)
Low birth weight at term^c^	2/177 (1.1)	145/8649 (1.7)	0.7 (0.2–2.7)	0.6 (0.2–2.6)
Stillbirth^d^	1/192 (0.5)	35/9247 (0.4)	1.4 (0.2–10.1)	2.0 (0.3–15.4)
Abnormalities^e^	11/181 (6.1)	323/8673 (3.7)	1.7 (0.9–3.1)	1.7 (0.9–3.1)
				
*Births in group 2*	(*N*=15)	(*N*=706)		
Preterm birth	8/15 (53.3)	30/704 (4.3)	25.7 (8.7–75.4)	26.6 (8.5–83.0)
Low birth weight at term^c^	0/7 (0.0)	9/674 (1.3)	—	—
Stillbirth^d^	0/15 (0.0)	2/706 (0.3)	—	—
Abnormalities^e^	1/13 (7.7)	18/606 (3.0)	2.7 (0.3–22.1)	2.7 (0.3–22.8)
				
*Births in group 3*	(*N*=85)	(*N*=4089)		
Preterm birth	5/85 (5.9)	205/4080 (5.0)	1.2 (0.5–2.9)	1.2 (0.5–2.9)
Low birth weight at term^c^	0/80 (0.0)	48/3866 (1.2)	—	—
Stillbirth^d^	0/85 (0.0)	9/4089 (0.2)	—	—
Abnormalities^e^	4/78 (5.1)	124/3742 (3.3)	1.6 (0.6–4.4)	1.6 (0.6–4.5)

Abbreviation: CI=confidence interval.

Group 1: birth outcome in women diagnosed with Hodgkin's disease before pregnancy.

Group 2: birth outcome in women diagnosed with Hodgkin's disease during pregnancy.

Group 3: birth outcome in women diagnosed with Hodgkin's disease within 2 years postpartum.

a Controlled for month and year of the birth and maternal county of residence (by matching).

b Further adjusted for maternal age (<25, 25–29, 30–34, and ⩾35 years) and parity (1 and 2+) by logistic regression. Calendar period of the birth (1973–1986, 1987–1994, and 1995–2002) was also included as an independent variable in the model.

c Preterm births were excluded from the analyses of low birth weight at term.

d Stillborn babies were excluded from the analyses of preterm birth, low birth weight at term, and congenital abnormalities.

e Data on congenital abnormalities included births from 1977 to 2002.

**Table 3:** Congenital abnormalities diagnosed during the first year of life in children of Hodgkin's disease patients

**Congenital abnormalities (CAs) according to organ system**	**Children**	**Type of congenital abnormality according to ICD-8/ICD-10**
*Group 1*	(*N*=11)	(*N*=13)
CA in cardiovascular organs	Child 1	Defectus septi atriorum cordis (Q21.1)
CA in respiratory organs	Child 2	CA in larynx (not specified) (Q31.8)
CA in urologic organs	Child 3	Polycystic, dysplastic kidney (Q61.4)
CA in bones and muscles		
Foot	Child 4	Pes planus congenitus (Q66.5)
Head, spine, and chest	Child 5 (first CA)	Pectus excavatum (Q67.6)
Other CA in bones and muscles	Child 6	Torticollis congenita (756.81)
Other CA in limbs	Child 7, 8, and 9	CA in limb (not specified) (Q74.9) (755.99)
Other CA in bones of skull and face	Child 10	CA in bone of skull and face (not specified) (Q75.9)
CA in muscle and bones,	Child 5 (second CA)	Hernia diaphragmatica congenita (Q79.0)
Not otherwise classified	Child 11 (first CA)	CA in muscle and bone (not specified) (Q79.8)
Other CA	Child 11 (second CA)	CA (not specified) (Q89.9)
		
*Group 2*	(*N*=1)	(*N*=2)
CA in cardiovascular organs	Child 1 (first CA)	Defectus septi atriorum cordis (Q21.1)
Chromosomal abnormality	Child 1 (second CA)	Down's syndrome (Q90.9)
		
*Group 3*	(*N*=4)	(*N*=11)
CA in cardiovascular organs	Child 1 (first CA)	Tetralogia Steno-Fallot (74.629)
	Child 1 (second CA)	CA in heart (not specified) (74.699)
	Child 2 (first CA)	Tetralogia Steno-Fallot (74.629)
	Child 2 (second CA)	CA in heart (not specified) (74.699)
	Child 3 (first CA)	Defectus congenitus septi ventricolorum (74.639)
	Child 3 (second CA)	Defectus congenitus septi atriorum (74.641)
	Child 3 (third CA)	Other specified CA in heart (74.689)
	Child 3 (fourth CA)	Coarctatio aortae (74.719)
	Child 3 (fifth CA)	Transpositio vasorum (74.619)
CA in bones and muscles foot	Child 1 (third CA)	Pes equino-varus (75.400)
	Child 4	Pes calcaneo-valgus (75.402)

Group 1: birth outcome in women diagnosed with Hodgkin's disease before pregnancy.

Group 2: birth outcome in women diagnosed with Hodgkin's disease during pregnancy.

Group 3: birth outcome in women diagnosed with Hodgkin's disease within 2 years postpartum.

**Table 4:** Birth outcome stratified by treatment with radiotherapy and calendar period of Hodgkin's disease diagnosis for women diagnosed with Hodgkin's disease before pregnancy (group 1)

	**Preterm birth**		**Low birth weight at term**		**Stillbirth**		**Abnormalities^a^**	
***N*=192**	**Outcome/total (%)**	**Adjusted POR^b^ (95% CI)**	**Outcome/total (%)**	**Adjusted POR^b^ (95% CI)**	**Outcome/total (%)**	**Adjusted POR^b^ (95% CI)**	**Outcome/total (%)**	**Adjusted POR^b^ (95% CI)**
*Radiotherapy* c								
Yes (*N*=100)	4/99 (4.0)	0.7 (0.2–1.8)	1/93 (1.1)	0.5 (0.1–3.6)	1/100 (1.0)	4.6 (0.5–38.5)	4/90 (4.4)	1.2 (0.4–3.3)
No (*N*=88)	8/88 (9.1)	1.8 (0.8–3.7)	1/80 (1.3)	0.9 (0.1–6.5)	0/88 (0.0)	—	6/87 (6.9)	1.9 (0.8–4.4)
								
*Time of Hodgkin's disease diagnosis*								
1970–1980 (*N*=66)	4/65 (6.1)	1.0 (reference)	0/60 (0.0)	1.0 (reference)	1/66 (1.5)	1.0 (reference)	1/55 (1.8)	1.0 (reference)
1981–1990 (*N*=64)	2/64 (3.1)	0.6 (0.1–2.4)	2/62 (3.2)	1.9 (0.5–8.1)	0/64 (0.0)	—	3/64 (4.7)	1.4 (0.4–4.4)^d^
1991–2000 (*N*=62)	6/62 (9.7)	1.8 (0.8–4.1)	0/55 (0.0)	—	0/62 (0.0)	—	7/62 (11.3)	3.1 (1.4–6.9)^d^

Abbreviations: CI=confidence interval; POR=prevalence odds ratio.

a Data on congenital abnormalities included births from 1977 to 2002.

b Controlled for month and year of birth and maternal county of residence by matching and further adjusted for maternal age and parity by logistic regression. Calendar period of the birth was also included as an independent variable in the model. Stillborn children were excluded from the analyses of preterm birth, low birth weight at term, and congenital abnormalities.

c Data on radiotherapy were missing for four women.

d Wald *χ*^2^-test, *P*=0.25.
